# Dosimetric and volumetric changes in the rectum and bladder in patients receiving CBCT‐guided prostate IMRT: analysis based on daily CBCT dose calculation

**DOI:** 10.1120/jacmp.v17i6.6207

**Published:** 2016-11-08

**Authors:** David Pearson, Sukhdeep K. Gill, Nina Campbell, Krishna Reddy

**Affiliations:** ^1^ Department of Radiation Oncology University of Toledo Toledo OH USA; ^2^ Department of Physics University of Salford Salford UK

**Keywords:** IGRT, prostate, CBCT, bladder, rectum, DVH

## Abstract

Delivered dose can be calculated by transferring the planned treatment beams onto the daily CBCT. Bladder and rectum volumetric doses were calculated and correlated to the daily bladder and rectum fullness. Patients for this study underwent hypofractionated prostate IMRT to 70 Gy in 28 fractions. Daily CBCT was utilized for image guidance. A clinically acceptable plan was created using a CTV‐to‐PTV uniform margin of 5 mm. Image fusion was performed to transfer the bladder and rectum contours onto each CBCT. Contours were then edited to match the anatomy of each CBCT. Using the daily treatment isocenter, the planned beams were transferred onto the CBCT and daily and cumulative DVHs calculated. For the results a total of 168 daily CBCTs were evaluated. The bladder was found to be smaller for 74.7% of the 168 daily CBCTs accessed in this study. This reduction in volume correlated to an increase in the cumulative bladder V70 Gy from 9.47% on the planning CT to 10.99% during treatment. V70Gy for the rectum was 7.27% on the planning CT, when all six patients were averaged, and increased to 11.56% on the average of all daily treatment CBCTs. Increases in volumetric rectum dose correlated with increases in rectal volume. For one patient, the rectum and bladder absolute V70 Gy, averaged over the course of treatment, increased by 295% and 61%, respectively. Larger variations in the daily bladder and rectal volume were observed and these correlated to large deviations from the volumetric dose received by these structures. In summary, bladder and rectum volume changes during treatment have an effect on the cumulative dose received by these organs. It was observed that the volumetric dose received by the bladder decreases as the volume of the bladder increases. The inverse was true for the rectum.

PACS number(s): 87.55.dk‐, 87.57.Q‐

## I. INTRODUCTION

Prostate cancer is the most commonly diagnosed male cancer worldwide, excluding skin cancer.^(1)^ Intensity‐modulated radiation therapy (IMRT) can be used to deliver high radiation doses to the prostate while improving local control. Image‐guided radiation therapy (IGRT) is used in the treatment of prostate cancer to assist with precise dose delivery to the tumor and to maximize the sparing of normal structures. Cone‐beam computed tomography (CBCT) can provide information on the day‐to‐day variation in the shape and size of the prostate, bladder, and rectum. Variations in the daily setup and volumes of the bladder and rectum can significantly alter the dose distribution in these organs, thus affecting the probability of late radiation‐related toxicities.^(2)^

Dosimetric studies ^3^, ^4^, ^5^ have shown the change in bladder and rectum volume during radiation therapy and the impact of it on delivered doses. McParland et al.^(6)^ in a study of five patients with prostate radiotherapy also observed that there was a significant difference in bladder and rectum volumes and doses as compared to the planning CT volumes and doses. Similarly, Hatton et al.^(7)^ also observed the difference in bladder and rectal volumes and doses in patients undergoing prostate radiotherapy. Landoni et al.^(8)^ have also studied the effect of setup errors and organ motion on prostate cancer IMRT and the results have proved that though rectum dose is very different than planned, it has negligible effect on prostate dose coverage.

The goal of this study is to analyze and quantify the actual dose received by the bladder and rectum during an entire course of radiotherapy with respect to daily changes in the shape and volume of these organs. In this study, daily CBCT data are used to investigate fluctuations in the delivered dose distributions as compared to the planned dose distribution, due to the patient's daily anatomy and setup uncertainties. The prostate and OAR were contoured on the daily CBCT that were taken before each day of treatment, and the actual dose delivered to these organs was assessed based on the patient's anatomy of the day. The delivered dose could be calculated by transferring the planned beams onto the daily CBCT. The bladder and rectum volumetric doses were calculated and correlated to their corresponding volumes. A single planning CT does not represent the whole course of radiation therapy for each patient; this study shows the difference between the planned and delivered rectum and bladder doses, as calculated on daily kV‐CBCTs.

## II. MATERIALS AND METHODS

### A. Patient cohort and CT planning

The study cohort consisted of 168 datasets of six patients diagnosed with low‐ and intermediate‐risk prostate carcinoma with no lymph‐node involvement. Planning CT images were acquired using a Brilliance CT Big Bore (Philips Healthcare, Bothell, WA). All patients were scanned in supine position with a knee wedge and vacuum bag for immobilization. CT scans were acquired from top of the iliac crest to 7 cm below the ischium using a 3 mm slice thickness. For each patient, a clinically acceptable plan with a PTV margin of 5 mm was generated using Pinnacle^3^ 9.6, a treatment planning system (Koninklijke Philips, N V, Amsterdam, The Netherlands). All patients were planned using 9 fields with gantry angles of 0°, 40°, 80°, 120°, 160°, 200°, 240°, 280°, and 320°, using a 10 MV photon beam IMRT technique using direct machine parameter optimization (DMPO). The prescription dose was 70 Gy in 28 fractions, with 2.5 Gy per fraction. For all plans it was ensured that more than 95% of the PTV was receiving the full prescription dose. The entire CTV was covered by 100% of the prescription dose. For OAR, the constraints were decided to be less than the Radiation Therapy Oncology Group (RTOG) 0815 guidelines.^(9)^ The passing criteria for the rectum was V70 Gy<10%,V60 Gy<25% and V70 Gy < 10 cc. Similarly, for the bladder: V70 Gy<10%,V65 Gy<20%. Lastly, for the femoral heads, V50 Gy<5% and V35 Gy<15% were accepted for plan approval. The definition of these dosimetric parameters is taken as the percent volume or absolute volume in cc covered by the dose given in Gray.

### B. CBCT acquisition

All patients were treated on a TrueBeam linear accelerator (Varian, Palo Alto, CA). For initial setup, simulation mark and alignment lasers were used. Before each treatment, daily verification imaging of the pelvis was performed by using on‐board imaging. The CBCT images were acquired using the standard “pelvis” mode settings of 125 kV, 80 mA, 13 ms, and full scan with half‐fan bowtie filter. An automatic match algorithm was used for the initial assessment of CBCT images and further verification was completed by a physician using manual matching. It was ensured that none of the CBCTs used in this study were clipped and missing patient tissue. This was necessary to avoid errors in dose calculations on the CBCTs.

### C. Contouring on CBCT

Daily CBCTs of all patients were exported from the “offline review” module in Varian ARIA (record and verify system) to MIM Maestro contouring software (MIM Software, Cleveland, OH). All contours and treatment isocenters were transferred from the planning CT to the daily CBCT using registration files. A rigid fusion was performed, using MIM, between the planning CT and daily CBCT using the translational shifts. The CTV (contoured as the prostate) was adjusted based on the daily anatomy, while the rectum, bladder, and femoral heads were recontoured according to the patient's anatomy of the day. Volumes of the rectum and bladder on daily CBCT, as compared to planning CT, are shown in Table 1.

Contouring was outlined according to the RTOG guidelines^(10)^ for a male pelvis. To reduce the interuser variability for contouring, the same investigator completed all contouring per patient.

**Table 1:** Bladder and rectal volume data

*P‐CT*		*Daily CBCT*		
	*p‐CT Volume*	*Mean*	*Minimum*	*Maximum*
*Bladder Volume (cc)*				
Patient 1	470.66	230.57	76.16	380.80
Patient 2	69.89	91.30	64.49	133.06
Patient 3	191.89	171.90	119.10	390.20
Patient 4	75.36	128.61	50.66	373.51
Patient 5	78.05	102.60	49.37	207.74
Patient 6	83.60	107.61	74.91	175.34
*Rectum Volume (cc)*				
Patient 1	41.50	58.09	47.50	93.50
Patient 2	83.40	92.17	69.66	109.69
Patient 3	78.59	84.79	60.00	121.08
Patient 4	76.59	84.26	68.84	110.19
Patient 5	154.50	147.61	101.04	219.29
Patient 6	63.62	49.54	31.29	79.42

### D. Dose calculation on CBCT

**Figure 1:**
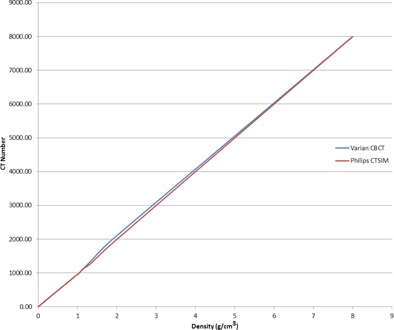
CT‐to‐density calibration curve for Varian CBCT vs. Philips CTSIM.

The CBCTs were transferred from MIM Maestro to the Pinnacle^3^ 9.6 treatment planning system, at which point the plan for each patient was transferred from the planning CT to the daily CBCT treatment isocenter. Plans were first transferred to RadCalc (LifeLine Software, Austin, TX), which is primarily a MU second‐check software package, and then transferred onto the daily CBCT using a script file. The script file generated was executed in Pinnacle. This allowed us to transfer beams from the original dataset to the CBCT datasets. The total MU, weightage for each beam, and the beam arrangement were kept the same as for the planning CT on daily treatment isocenter. For each patient, daily doses were calculated as per anatomy of the day. For dose calculation accuracy, an anatomical site‐specific CT number‐to‐density calibration curve for our CBCT was used. The results of the CT number‐to‐density curve for the Philips Gemini CT (Philps Healthcare, Bothell, WA) and Varian OBI were in good agreement, as shown in Fig. 1. Using the daily treatment isocenter, the planned beams were transferred onto the CBCT, and the daily and cumulative DVHs were calculated. Raw data from Pinnacle were exported to Excel spreadsheets using script files.^(11)^ This process was used to calculate dosimetric parameters and DVHs for each daily CBCT for each patient. Cumulative dosimetric parameters where also calculated by taking the average of each parameter, and its associated standard deviation, for all 168 datasets. For example, when considering V70 (cc) for the rectum, all 168 V70 (cc) values were averaged leading to a cumulative V70 (cc).

## III. RESULTS

Daily CBCTs were used from six patients who underwent image‐guided IMRT for localized carcinoma prostate, resulting in a total of 168 CBCT datasets used for analysis.

### A. CTV coverage

For the planned dose, the CTV V100 for all six patients was 100%, and the cumulative V100, found by averaging over all CBCTs, was 95.53%±10.17% (V100%+SD). Similarly, V95 and V90 were 99.95%±0.21% and 99.99%±0.07%, respectively. For the uniform 5 mm PTV margin used in this study, the CTV was covered by a 95% isodose line on 99.95% of daily treatments when averaged over all 168 treatments.

### B. Bladder volume changes and dosimetric variations

The bladder volume for six patients for all daily CBCTs, as compared to the planning CT, was calculated. For one patient, the volume of the bladder on the planning CT was 470.6 cc, but the minimum and maximum volumes when averaging over all CBCTs were 76.16 cc and 380 cc. The mean bladder volume for the same patient over the 28 CBCTs was 230.5 cc.

**Figure 2:**
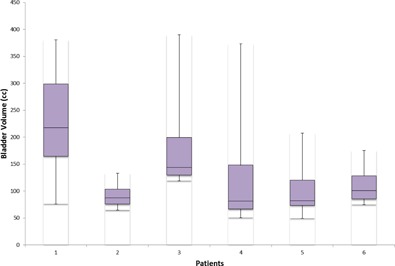
Variations in the bladder volume contoured on CBCT for six patients.

**Figure 3:**
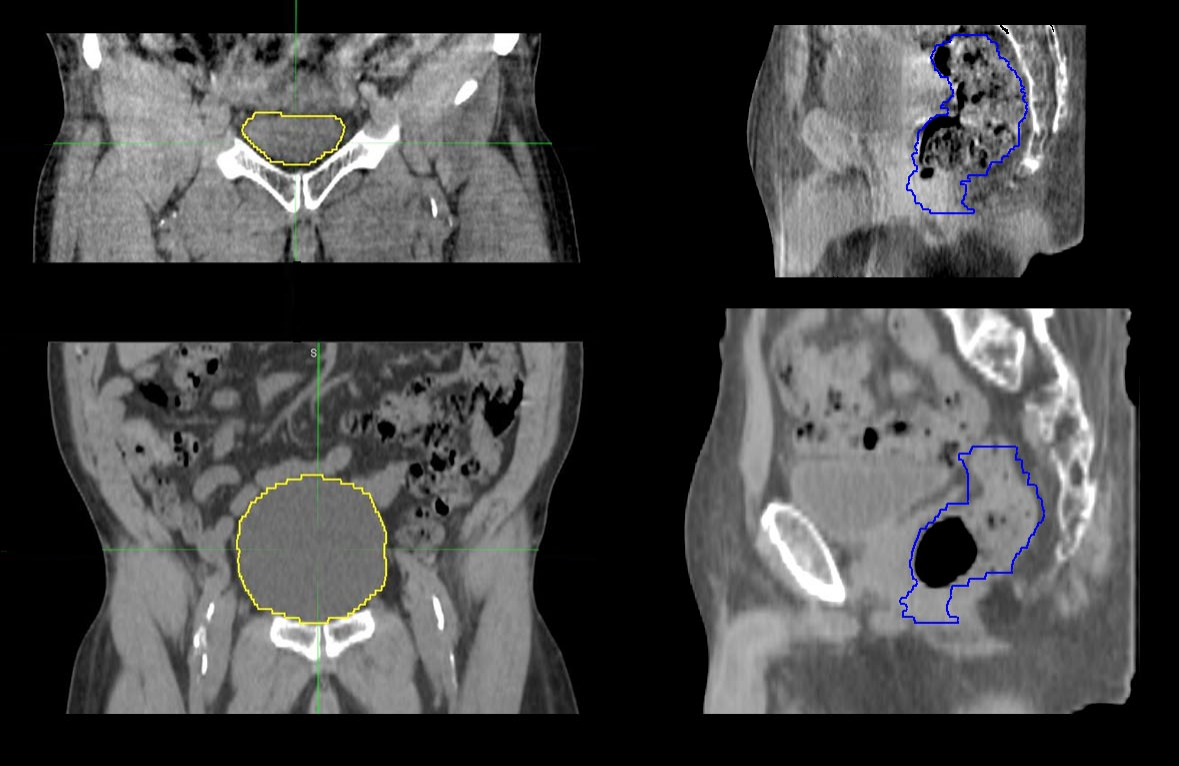
Change in bladder (left) and rectum (right) volume on P‐CT (bottom) and daily CBCT.

To graphically express how bladder volume varies during an entire course of treatment, Fig. 2 shows the bladder volumes for all six patients as per daily CBCT. The box in the plot shows median, lower‐, and upper‐quartile range and the whiskers represent the outliers. The change in planned versus daily CBCT volume for one of the patient is highlighted in Fig. 3, thus agreeing with literature.^12^

**Figure 4:**
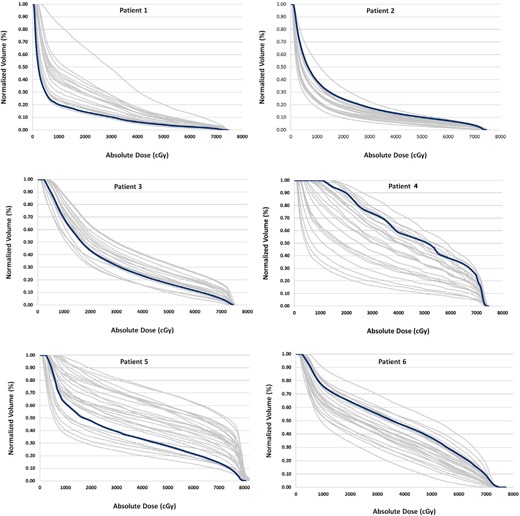
Volumetric dose comparison of bladder on daily CBCT (gray) and planning CT (blue) for six patients.

Variations in the volume of the bladder during a course of radiation therapy directly altered the dose received by the bladder. A comparison of the bladder dose on the planned and cumulative CBCTs is presented in Table 2. Here, results show that the average volumetric doses received by the bladder were in good agreement with the planned doses. As shown in Table 3, the average V70 (cc) for the planned dose for all patients is 9.91 cc and the average cumulative V70 (cc) for all patients on the daily CBCT is 11.67 cc with a standard deviation of 4.53 cc. Daily variations were averaged for all CBCTs. The cumulative planned V70 Gy averaged over all patients was 9.47%. Cumulative V70 Gy for all daily CBCTs was 10.99% with a standard deviation of 11.71%, which exemplifies the variation of the bladder volume throughout a course of treatment. The considerable day to day variations can also be seen by the bladder DVHs in Fig. 4. To highlight the dosimetric effects of these large variations in bladder volume let us consider the example of Patient 5, with an average bladder volume of 102.6 cc and a minimum volume of 49.37 cc. The planned bladder V70 Gy was 17.34% and the average for all treatments 27.72%±10.86%. However, on the day when the bladder was at a minimum volume of 49.37 cc, the bladder V70 Gy rose to 54% — more than three times the planned dose.

**Table 2:** Rectum and bladder dose comparison. Cumulative CBCT refers to the dosimetric parameters found by averaging over all 168 CBCTs for all patients

	*Rectum Dose (in %)*		*Bladder Dose (in %)*	
*Dose*	*Planned*	*Cum*. CBCT±SD	*Planned*	*Cum*. CBCT±SD
V70 Gy	7.27	11.56±5.65	9.47	10.99±11.71
V65 Gy	12.90	16.83±6.94	14.27	14.98±13.13
V60 Gy	17.58	20.80±7.83	17.53	18.03±14.34
V50 Gy	25.84	27.77±9.19	24.54	23.82±16.59
V40 Gy	33.88	34.88±10.43	30.16	29.82±18.59
V30 Gy	43.11	43.66±13.09	37.96	37.01±20.48

**Table 3:** Rectal and bladder V70 (cc) comparison in planning and daily CBCT

	*Rectum V70 (cc)*		*Bladder V70 (cc)*	
	*P‐CT*	*Daily* CBCT±SD	*P‐CT*	*Daily* CBCT±SD
Patient 1	2.50	8.17±3.24	4.64	4.97±1.80
Patient 2	3.07	7.78±2.93	2.59	2.30±0.77
Patient 3	4.17	16.49±5.39	9.50	15.30±8.52
Patient 4	9.56	8.18±2.86	17.82	13.90±2.30
Patient 5	17.37	11.50±6.23	17.34	27.72±10.86
Patient 6	2.74	5.51±2.72	7.56	5.86±2.95

DVH comparison of the planning CT and daily CBCT for the whole course of treatment for six patients is shown in Fig. 4. On average, for all six patients, 74.7% of the time the daily CBCT bladder volume is less than the planned bladder volume. For Patient 1 in Fig. 4, 100% of the time the daily CBCT volume is smaller than the planned CT volume. The volume of the bladder on the planning CT is greater than the volume during the entire treatment, meaning that the patient is receiving a higher dose than expected during all days of treatment. As another example, for Patient 2, Fig. 4 shows that 92.8% of the time (or 26 out of 28 treatments) the daily CBCT bladder volume is larger than the planned volume. As shown by the DVH for each patient, the received bladder dose is significantly different form the planned dose. The delivered average V70 of the bladder decreased for patients whose bladders where larger at the time of treatment that at planning. This was true for five of the six patients. Patient 5 had a mean bladder volume of 102.60 cc at treatment, compared to 78.05 cc on the planning CT, but the average V70 for treatment was greater despite the larger bladder volume.

### C. Rectal volume changes and dosimetric variations

**Figure 5:**
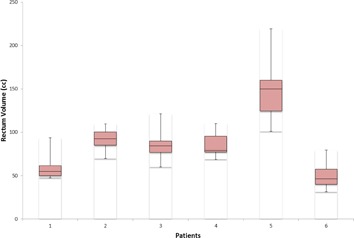
Variations in the rectum volume contoured on CBCT for six patients.

Rectum volume also varied significantly on a daily basis, so the actual dose received by the rectum was different in comparison to the planned dose. Average, minimum, and maximum rectal volumes for each patient as compared to planned CT rectal volume are shown in Table 1. For a 5 mm isotropic CTV‐to‐PTV margin, the planned CT V70 Gy was 7.27%, but the cumulative V70 Gy average when considering all CBCTs was 11.56%, with a 5.65% standard deviation. Similarly, the planned V65 Gy was 12.9% as compared to the cumulative average of 16.83%, with 6.94% standard deviation. Figure 5 shows the daily rectum volume variations for all six patients.

**Figure 6:**
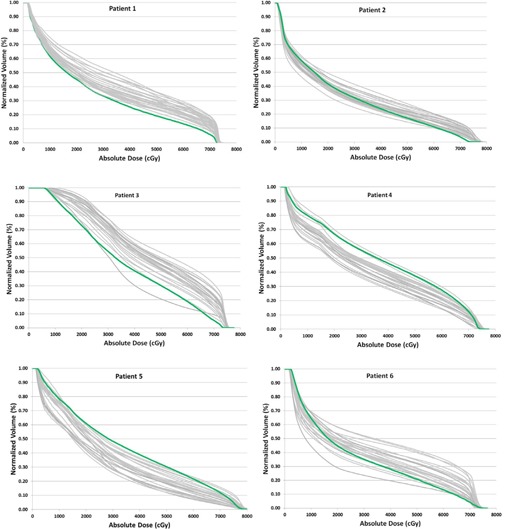
Volumetric dose comparison for rectum on daily CBCT (gray) and planning CT (green) for six patients.

The average rectal volume on the planning CT for all six patients was 83.03 cc, whereas the average daily CBCT rectal volume for all patients was 86.10 cc. So 45.2% of the time, the rectal volume on the daily CBCT was greater than on the planned CT. For V70 (cc), the average planned rectal volume for all patients was 6.57 cc, as compared to the average daily CBCT V70 (cc) of 9.48 cc, with a standard deviation of 5.32 cc. The comparison of the planned V70 (cc) and the daily CBCT V70 for each patient can be seen in Table 3, and a DVH comparison of the planned and daily CBCT rectal dose is shown in Fig. 6.

## IV. DISCUSSION

Daily setup variations and organ motion during prostate IMRT yield differences in the actual vs. expected dose received by the prostate, rectum, and bladder. Results of this study indicate that large variations occur in the bladder and rectal volume during each day of treatment.

Studies have shown the effect that variation in the bladder and rectum volumes has on prostate coverage. Changes in bladder volume allows for an increase in interfraction motion, hence affecting CTV coverage. This is a concern, particularly when studies such as Frank et al.^(12)^ have shown that 90% of time the bladder volume is found to be smaller than the planned volume.

Several studies have reported the change in bladder and rectal volume during the course of prostate radiotherapy. This has an impact on the radiation doses received to bladder and rectum and, thus, further radiation induced toxicities. Sripadam et al.^(13)^ have reported the volumetric change of rectum during treatment based on daily CBCT. The change in rectal volume during the course of radiotherapy was significant. In patients with low rectal volume at planning (less than 50 cc), up to 25% more rectal volume was included in the high‐dose region.

Kupelian et al.^(14)^ have reported the change in bladder and rectal doses based on daily MV CT calculations. Our study is actually taking into account for the dosimetric and volumetric change in each and every patient for daily treatment based on CBCT calculations. It has been reported that bladder and rectum volume changes can affect the CTV dose; however, CTV coverage in this study was not negatively affected by bladder and rectal volume change. This may indicate that our CBCT‐based patient alignment strategy, combined with a uniform 5 mm PTV margin, is sufficient to compensate for daily variations.

Due to the daily variations in the bladder position and shape, the bladder trigone might receive higher doses of radiation, which leads to further increased acute and late urinary toxicities. Ghadjhar et al.^(15)^ in a study of 268 patients of prostate radiotherapy showed that radiation dose to the trigone was significantly associated with late urinary toxicities.

It can be seen that variations in the bladder volume affect the amount of dose received, and so the importance of patients maintaining a stable bladder volume throughout a course of treatment is highlighted. The smaller the volume of the bladder, the greater the volumetric dose that is received. The CBCT‐generated DVHs have shown that not all the patients were able to maintain a stable rectal and bladder volume, which is one of the biggest challenges for prostate IMRT.

In 74.7% of the 168 treatments analyzed the bladder volume, on a daily basis, was less than the planned bladder volume. Daily rectal volumes showed a more even distribution, being greater than the planned rectal volume for 45% of the time. For the rectum, a lower volume led to a lower volumetric dose received. We have reported the changes in bladder and rectum volumetric, as well as dosimetric, and these changes are clinically significant.

Newer advances in form of endorectal balloons and transperineal inserted spacers have been discussed by Smeenk et al.^(16)^ and are used in prostate radiotherapy to decrease the rectal toxicity. Endorectal balloons help in maintaining the consistent volume of rectum during the course of prostate radiotherapy and thus the variation in radiation dose received to the rectum is reduced. Similarly, strict bladder protocols can result in almost‐consistent volume of the bladder daily, thus decreasing the variation in the radiation dose received to the bladder.

Finally, it is understood that there are known issues associated with the use of CBCT for daily dose calculations.^(17)^ In pelvic phantom studies,^(18)^ when using a site‐specific CT number for the density calibration of CBCT images, a 2% dose accuracy agreement has been observed between the planning CT and CBCT images. In our study, an anatomical site‐specific CT number to density calibration curve for CBCT dose calculation was used and our results between CT and daily CBCT are in a good agreement.^(19)^ In our clinical workflow, doses from the daily CBCTs are not counted towards the patient's prescribed dose. For this reason the daily CBCT was not accounted for in this study.

Although there are studies focused on rectum or bladder volume changes, in the present study the actual dose delivered to the prostate, rectum, and bladder is computed volumetrically. This work is unique in that each daily CBCT was used to produce a daily DVH, volumes were accurately tabulated based on 168 CBCT datasets, and the dose delivered to each structure was calculated with a minimum number of assumptions made.

## V. CONCLUSIONS

Volumetric dose received by the rectum and bladder differ significantly as compared to planned dose due to changes in the shape of size of these organs. As the volume of the bladder increases, the dose received by the bladder decreases. However, as the size of rectum increases, so does the rectal dose. The magnitude of the dosimetric effects can be significant and therefore it is strongly recommended that bladder and rectum volume should be kept consistent, or as close as possible, on a daily basis, for patients undergoing treatment.

## COPYRIGHT

This work is licensed under a Creative Commons Attribution 3.0 Unported License.
